# The Association of Drug-Use Characteristics and Active Coping Styles With Positive Affect in Patients With Heroin-Use Disorder and Methamphetamine-Use Disorder During the COVID-19 Pandemic

**DOI:** 10.3389/fpubh.2021.739068

**Published:** 2021-12-03

**Authors:** Yingying Wang, Jinsong Zuo, Long Wang, Qianjin Wang, Xin Wang, Qian Yang, Hanjing Emily Wu, Colin B. Goodman, Dongmei Wang, Tieqiao Liu, Xiangyang Zhang

**Affiliations:** ^1^Department of Psychiatry, The Second Xiangya Hospital of Central South University, Changsha, China; ^2^National Clinical Research Center for Mental Disorders, The Second Xiangya Hospital of Central South University, Changsha, China; ^3^School of Life Science and Chemistry, Hunan University of Technology, Zhuzhou, China; ^4^Sanming Taijiang Hospital, Sanming, China; ^5^Department of Psychiatry and Behavioral Sciences, The University of Texas Health Science Center at Houston, Houston, TX, United States; ^6^CAS Key Laboratory of Mental Health, Institute of Psychology, Chinese Academy of Sciences, Beijing, China

**Keywords:** COVID-19, substance use disorders, positive affect, withdrawal, craving

## Abstract

**Background:** Positive affect (PA) is crucial for individuals to cope with the current pandemic and buffer the lingering fears after it, especially for patients with substance-use disorders (SUDs). The current study aimed to explore PA and its related factors during the COVID-19 pandemic in male patients with the heroin-use disorder (HUD) and patients with the methamphetamine-use disorder (MAUD), respectively.

**Methods:** A total of 325 male patients with SUDs (106 with HUD and 219 with MAUD, all were single-substance users) in a compulsory rehabilitation center underwent semi-structured interviews during the pandemic. The demographic information, drug-use characteristics, active coping styles (ACSs, by Simple Coping Style Questionnaire), and PA (by the Positive and Negative Affect Scale) of participants were collected and recorded.

**Results:** There were significant differences between the two groups in age, the proportion of full-time workers before the epidemic, duration of drug use, the proportion of patients with long-term withdrawal during the epidemic, cravings, ACS, and PA. Correlation and multiple linear regression analysis showed that duration of drug use, ACS, and stable jobs were significant predictive factors for PA in patients with HUD, while long-term withdrawal, ACS, and stable jobs during the epidemic were significant predictive factors for PA in patients with MAUD.

**Conclusions:** Our study demonstrated the factors for PA in patients with HUD and MAUD during the pandemic. The results provided a basis for the comprehensive understanding of the PA of patients with SUDs and the development of targeted treatments.

## Introduction

The outbreak of COVID-19 has caught people off guard globally (1). General public events, such as the COVID-19 pandemic, have had an impact on the physical and mental health worldwide of people (2, 3). The uncertain prognosis, shortage of testing and treatment resources, increasing economic losses, and negative effects of home confinement on physical health (4) have worked as a cluster of stressors and inevitably brought anxiety and depression to individuals (5–7), with affected populations being the elderlies (8, 9), children (10–12), teenagers with low awareness of risk for infection (13), college students receiving online courses (14), and pregnant women who are unable to access medical care due to home confinement (15). For some of those with existing mental health disorders (16–18), the COVID-19 pandemic has aggravated their conditions (19, 20). Several recent studies have shown that some individuals may resort to addictive behaviors to relieve their stress during the pandemic, particularly alcohol abuse (21) and internet-related addictions (22, 23). Some studies also indicated that the mental problems of patients with the substance-use disorder (SUDs) could relapse (24, 25) or progress (26, 27) during the pandemic due to the social isolation under lockdowns; in some severe cases, the patients take overdoses on their own (28). Moreover, patients with preexisting SUDs are at an increased risk for adverse outcomes following COVID-19 infection (29–32). Thus, these patients are under greater pressure in the face of the pandemic, which needs the attention of health authorities.

Having realized the significant impact of the COVID-19 pandemic, many researchers began to focus on affect problems related to the pandemic, which may provide a basis for timely mental health services during the pandemic (33). However, these studies focused more on negative affect (NA) rather than on positive affect (PA) (34). In fact, it has been demonstrated that PA also plays an important role in coping with chronic stressors through improving social, intellectual, and physical conditions of patients (34, 35). PA also counteracts negative physiological effects of chronic stressors and reduces the likelihood of post-traumatic depression (36, 37), indicating that it may help patients recover from NA related to the pandemic (35, 38). Moreover, PA is involved in information processing (39–41), which also reflects its importance regarding the high information load during the pandemic. PA can also alleviate the negative physiological consequences caused by stress (42, 43), which is beneficial to the physical conditions of individuals to defend against the coronavirus. To sum up, PA plays a more valuable role than most people think in coping with the pandemic (44). Of note, PA is an important factor for treatment outcomes in patients with SUDs (45–47), with suppressed PA associated with poorer outcomes (48) and improved PA associated with a better perception of quality of life (49, 50). In conclusion, clarifying the factors related to PA for patients with SUDs is conducive for them to face the pandemic positively. Some prior studies have shown that active coping styles (ACSs, such as seeking social support from others, engaging in physical activities, and positive reappraisal) are associated with PA in the general population (51–53), which is the same during the COVID-19 pandemic (54–56).

To date, heroin (an opioid substance) and methamphetamine (MA, a stimulant) are the most widely abused illegal drugs across the world, especially in Asia (57). Previous studies have found differences in several clinical aspects, such as demographics (58), personality traits (59), and the process of addiction (60) between patients with the heroin-use disorder (HUD) and patients with the methamphetamine-use disorder (MAUD). However, no studies have compared PA-related factors between the two disorders, especially in the context of COVID-19. Therefore, the present study aims to explore the factors and latent differences of PA between patients with HUD and those with MAUD. In addition to ACS mentioned above, we also included some characteristics of drug use, such as duration of drug use, long-term withdrawal (i.e., with no drug use for at least 3 months), and cravings, as potential factors during the COVID-19. Since the COVID-19 pandemic is a once-in-a-lifetime stressor, we also proposed some key considerations in demographics. In this study, we also aim to explore the differences in demographics and drug-use characteristics between two groups of patients with different SUDs and identify the factors of PA for the two SUDs.

## Methods

### Participants and Procedures

From July to September 2020, a total of 733 patients with SUDs (133 women and 600 men) admitted to a compulsory drug rehabilitation center (Changsha, Hunan Province, China) underwent semi-structured interviews by two trained psychiatrists. According to our aim, only 325 male patients with single HUD (*n* = 106) or MAUD (*n* = 215) were retained. The inclusion criteria were as follows: (1) patients diagnosed with HUD or MAUD based on DSM-5 and (2) with at least 2 weeks of withdrawal at the time of recruitment. The exclusion criteria were as follows: (1) patients diagnosed with other mental disorders, (2) with serious physical diseases, (3) with intellectual or cognitive impairment, and (4) who cannot understand the questionnaires.

This study was approved by the Ethics Committee of The Second Xiangya Hospital of Central South University. All the participants in the study provided written informed consent; they were informed that they could withdraw from the study at any time without needing to provide any reason, and all their information was confidential.

### Measures

A combination of semi-structured interviews and self-reports of patients were included in this study.

#### Semi-structured Assessment for Drug Dependence and Alcoholism (SSADDA)

For the screening of SUDs and other mental disorders, SSADDA was originally developed by Yale University (61, 62). It has been translated into different languages and verified for its reliability and validity in the SUDs population (63, 64). SSADDA was translated by our team in 2017 and was tested for psychometric properties, which indicated that the Chinese version of SSADDA had good reliability and validity when applied in patients with SUDs (65). SSADDA has two main functions: One is to diagnose SUDs based on DSM-5 (66), including the abuse of tobacco, alcohol, MA, ketamine, opioid, and other substance (such as marijuana); and the other function is to screen out other mental disorders, such as schizophrenia (67), ADHD (68), and depression (69). SSADDA also reflects the characteristics of substance use, such as the duration of drug use and frequency of most severe episodes (70), which can help psychiatrists take the drug-use history of subjects.

#### Self-Reported Characteristics of Drug Use During the COVID-19 Pandemic

The participants were asked two questions about the characteristics of drug use during the pandemic. The first question was “Since the beginning of the COVID-19 outbreak, have you used no substance at all for at least 3 months?” and “a long period of withdrawal” was recorded if the answer was “yes.” The model for the assessment of previous long-term withdrawal experience of patients was established after SSADDA. The second question was “Since the beginning of the COVID-19 outbreak, what is the highest level of your craving for the substance you use?” and the level should be reported by the subject with the use of the Visual Analog Scale of Craving (VASC). VASC is a line segment bisected with the numbers of 0–10, with the leftmost number “0” representing “no cravings at all” and the rightmost number “10” representing “very strong and almost uncontrollable cravings” (71, 72).

#### Active Coping Style

The Simplified Coping Style Questionnaire (SCSQ) was used to evaluate the coping styles of the subjects. SCSQ (73) is an instrument with good reliability and validity and has been widely used in studies in China, especially during the pandemic (74, 75). It consists of two subscales that measure active and negative coping styles of participants with a Likert 4-point scale, with 0 representing “never” to 3 representing “always”; higher scores indicated a higher frequency of adopting the corresponding coping styles. For the purpose of our study, we only analyzed the total score of the ACS subscale, which has a Cronbach coefficient of 0.860.

#### Positive Affect

The PA of the participants was measured using the Chinese version of the Positive and Negative Affect Scale (73, 76), which is widely used in a variety of populations, including patients with SUDs. The original scale includes two subscales, i.e., subscales for PA and NA, respectively, with each one containing 10 words that describe the corresponding affect (e.g., energetic, cheerful, or pride for PA, and nervous, irritable, or confused for NA) during a certain period. Each item was rated with a Likert 5-point scale, with 0 = hardly and 4 = extremely. As this study was focused on PA, only the PA subscale was used for the analysis; its Cronbach coefficient in this study was 0.887.

### Statistical Analysis

Independent-samples *t*-test was used to analyze the differences in demographic data, drug-use characteristics, ACS, and PA between the two groups of patients with SUDs. Pearson's correlation was then used to analyze the relationship between the above clinical variables and PA. Finally, multiple linear regression analysis was performed for the two groups, respectively. PA was set as the dependent variable, and all variables with *p* < 0.1 in the previous correlation analysis were included as independent variables. Data analyses were performed using the SPSS software (version 23.0), with a significance level of *p* < 0.05 (two-tailed).

## Results

### Comparison of Demographic Data Between Patients With HUD and Patients With MAUD

The demographic information of the two groups is presented in Table 1. Patients with HUD had significantly higher age than those with MAUD (*p* < 0.001) and significantly fewer years of education (*p* = 0.028). There was no significant difference in the marital (i.e., married, unmarried, or divorced) and employment status (i.e., full-time job, part-time job, or unemployed) between the two groups.

**Table 1:** Demographic information of patients with HUD and patients with MAUD.

**Variables**	**Patients with HUD**	**Patients with MAUD**	**χ^2^/*t***	***p*-value**
	***n* = 106**	***n* = 219**		
Age	48.95 (7.24)	35.08 (6.93)	−16.675	<0.001
Education (years)	9.40 (2.96)	10.21 (3.21)	2.210	0.028
**Marital status**				
Married	46 (43.4)	102 (46.6)	0.291	0.590
Unmarried/devoiced	60 (56.6)	117 (53.4)		
**Employment status**				
Enterprises/self-employed	34 (32.1)	76 (34.7)	0.220	0.639
Part-time work/unemployed	72 (67.9)	143 (65.3)		

*HUD, heroin-use disorder; MAUD, methamphetamine-use disorder*.

### Comparison of Clinical Variables Between Patients With HUD and Patients With MAUD

The drug-use characteristics, ACS, and PA of the two groups are presented in Table 2. Duration of drug use was significantly longer in patients with HUD than in patients with MAUD (*p* < 0.001). A significantly higher proportion of the patients with HUD had a long-term withdrawal during the COVID-19 pandemic, as compared with those with MAUD (*p* < 0.001); the cravings during the epidemic in patients with HUD were significantly greater than in those with MAUD (*p* < 0.001). The scores of ACS and PA of patients with HUD were significantly lower than those in patients with MAUD (both *p* < 0.001).

**Table 2:** Clinical variables of patients with HUD and patients with MAUD.

**Variables**	**Patients with HUD**	**Patients with MAUD**	**χ^2^/*t***	***p*-value**
	***n* = 106**	***n* = 219**		
Duration of drug use (year)	23.41 (8.12)	9.47 (4.67)	−16.4111	<0.001
**Long-term withdrawal during COVID-19**				
Yes	20 (18.9)	160 (73.9)	84.890	<0.001
No	86 (81.1)	59 (26.9)		
Cravings during COVID-19	4.84 (2.93)	2.67 (2.26)	−6.719	<0.001
Total score of ACS	18.29 (5.00)	21.32 (6.46)	4.644	<0.001
Total score of PA	22.02 (5.20)	28.29 (6.78)	9.191	<0.001

*HUD, heroin-use disorder; MAUD, methamphetamine-use disorder; ACS, active coping styles; PA, positive affect*.

### Correlation Between Clinical Variables and PA in Patients With HUD and Patients With MAUD

Variables associated with PA for both SUDs are presented in Table 3. In patients with HUD, age (*r* = −0.225, *p* = 0.020), employment status (*r* = 0.240, *p* = 0.013), duration of drug use (*r* = −0.300, *p* = 0.002), and ACS (*r* = 0.250, *p* = 0.010) were significantly correlated with PA. In patients with MAUD, age (*r* = −0.140, *p* = 0.038), employment status (*r* = 0.199, *p* = 0.003), long-term withdrawal during COVID-19 (*r* = 0.274, *p* < 0.001), craving during the epidemic (*r* = −0.220, *p* = 0.001), and ACS (*r* = −0.241, *p* < 0.001) were significantly associated with PA.

**Table 3:** Correlation between clinical variables and positive affect in the two groups of patients.

**Groups**	**Positive affect**	***p*-value**
**Patients with HUD (*****n*** **=** **106)**		
Age	−0.225	0.020
Education	−0.009	0.931
Marital status	−0.177	0.070
Employment status	0.240	0.013
Duration of drug use	−0.300	0.002
Long-term withdrawal	0.105	0.282
Cravings	0.052	0.596
ACS	0.250	0.010
**Patients with MAUD (*****n*** **=** **219)**		
Age	−0.140	0.038
Education	0.077	0.255
Marital status	−0.012	0.863
Employment status	0.199	0.003
Duration of drug use	0.009	0.896
Long-term withdrawal	0.274	<0.001
Cravings	−0.220	0.001
ACS	0.241	<0.001

*HUD, heroin-use disorder; MAUD, methamphetamine-use disorder; ACS, active coping style*.

### Multiple Linear Regression of Clinical Variables to PA in Patients With HUD and Patients With MAUD

Multiple linear regression analysis was performed in patients with HUD and MAUD, respectively. PA was set as the dependent variable, and variables with *p* < 0.1 in the previous correlation analysis were taken as independent variables. The results (see Table 4) showed that duration of drug use (β = −0.267, *t* = −2.954, *p* = 0.004), ACS (β = 0.204, *t* = −2.258, *p* = 0.026), and stable job (β = 0.201, *t* = 2.223, *p* = 0.028) were significant predictive factors for PA (*F* = 7.423, *p* < 0.001, adjusted *R*^2^ = 0.155) in patients with HUD, while long-term withdrawal during the pandemic (β = 0.251, *t* = 3.986, *p* < 0.001), ACS (β = 0.226, *t* = 3.604, *p* < 0.001), and stable job (β = 0.165, *t* = 2.612, *p* = 0.010) were significant predictive factors for PA (*F* = 13.240, *p* < 0.001, adjusted *R*^2^ = 0.144) in patients with MAUD.

**Table 4:** Multiple linear regression of clinical variables to positive affect in the two groups of patients.

**Predictors**	**β**	***t***	***p*-value**	***F***	**Adjusted *R*^2^**
**Patients with HUD (*****n*** **=** **106)**					
Duration of drug use	−0.267	−2.954	0.004	7.423^***^	0.155
ACS	0.204	−2.258	0.026		
Stable job	0.201	2.223	0.028		
**Patients with MAUD (*****n*** **=** **219)**					
Long-term withdrawal	0.251	3.986	<0.001	13.240^***^	0.144
ACS	0.226	3.604	<0.001		
Stable job	0.165	2.612	0.010		

*** *p < 0.001; HUD, heroin-use disorder; MAUD, methamphetamine-use disorder; ACS, active coping style*.

## Discussion

To our knowledge, this is the first study to examine PA in patients with HUD and patients with MAUD during the COVID-19 pandemic. The results showed significant differences in age, education, some drug-use characteristics (i.e., duration of drug use, long-term withdrawal, and cravings during the pandemic), ACS, and PA between the two groups. Correlation analysis showed that age, employment status, duration of drug use, and ACS were significantly associated with PA in patients with HUD, while age, employment status, long-term withdrawal during the pandemic, cravings during the pandemic, and ACS were significantly associated with PA in patients with MAUD. Multiple linear regression analysis indicated that the duration of drug use, ACS, and stable job were significant predictive factors for PA in patients with HUD, accounting for 15.5% of the variation; long-term withdrawal, ACS, and stable job were significant predictive factors for PA in patients with MAUD, accounting for 14.4% of the variation.

With regard to demographics, patients with HUD were at a significantly higher age than those with MAUD, which was consistent with previous studies (59). In our study, the mean age of patients with HUD was nearly 50 years, and the duration of heroin use for this group was 23.41(±8.12) years, which is equivalent to the elderly stage of the life cycle in patients with HUD (77), indicating the advanced age of this group. As a result, they are a vulnerable group to both physical and psychological problems (78) and need the attention of healthcare providers. The level of education in patients with HUD was significantly lower, which might be a barrier for these patients to gain knowledge of COVID-19; this was in line with some previous studies, which showed that people with low education levels scored low in surveys regarding the knowledge of COVID-19 (79). With regard to drug-use characteristics, the duration of drug use in patients with HUD was significantly longer than that in patients with MAUD, which is consistent with the fact that their age was highly correlated with the duration of drug use (59, 80). In general, the older patients were more vulnerable to physical illnesses as they had long-term use of harmful substances (81, 82), which may increase their risk for infection with COVID-19. During the pandemic, 73.9% of the patients with MAUD had a withdrawal for more than 3 months, while the percentage was only 26.9% in patients with HUD. A possible reason for this significant difference is that MA might be harder to get; according to a survey, the amount of MA seized by the police significantly decreased through April 2020, while the seizure of heroin remained unchanged (83). Furthermore, patients with HUD are often highly addicted to heroin, meaning that they are less likely to withdraw and more likely to relapse (84). Moreover, our study also found that patients with HUD had significantly stronger cravings than those with MAUD during the pandemic, indicating that the level of cravings is also a risk factor for drug withdrawal (85). Patients with HUD had significantly higher ASC scores than those with MAUD, indicating that the former had adopted more ACS during the epidemic. Finally, as compared to patients with HUD, those with MAUD scored higher in PA. A possible reason for this difference is that the patients with HUD were at a higher age. Previous studies have shown that elderlies usually have lower levels of PA than younger people due to their reduced daily activity (86–88) and chronic illnesses (89, 90). This might be related to the reduced ability to perceive PA in patients with HUD due to the damage of corresponding brain regions (91, 92). Our results reflected that the biological mechanisms that produce PA in patients with HUD are even more impaired, i.e., their PA is less likely to be aroused than users of stimulants in the face of stressors. Therefore, treatment with regard to biological mechanisms for such patients is needed in response to the pandemic.

Correlation and multiple linear regression analysis revealed a slight difference in predictive variables for patients with HUD and patients with MAUD. First, the duration of drug use was a predictive factor for PA in patients with HUD only, whereas long-term withdrawal during the epidemic was a predictive factor for PA in patients with MAUD only. This suggested that although drug-use characteristics are important factors for patients with SUDs, their effects may vary on patients using different substances. A significant finding of this study was that long-term withdrawal was a protective factor for PA in patients with MAUD. Prior studies on the mechanism showed that the processing ability of PA recovered with the withdrawal of patients with SUDs (93, 94), which was conducive to their outcomes (95). Although some researchers suggested that lockdown-induced withdrawal might not be voluntary for those patients with SUDs, our results still showed the benefit of passive withdrawal due to inaccessibility to illicit drugs. Of note, the two groups shared two common predictive factors, one of which was the pre-pandemic employment status and the other was their ACS. As lockdowns led to some unemployment, the employment status of patients before the pandemic has become another point worth exploring. Studies showed that people who had long commutes for work or part-time or casual workers, such as migrant workers and retailers, are more likely to lose their jobs (96, 97), suggesting that they might be worse off under the stress of the pandemic compared to those with a secured job. This is in line with our results, which demonstrated that patients with stable jobs (e.g., employees of an enterprise or self-employers) had higher levels of PA than those with unstable jobs (e.g., casual workers or unemployed people). This might be due to the less financial pressure for those with stable jobs and who were more able to afford drugs and medical services they needed.

As mentioned above, ACS is positively correlated with PA (98, 99), which is consistent with our results. Due to the lockdowns, many people were confined to their homes (100, 101) and had to reduce activities and communication with others (102), which had an impact on those who were more dependent on others or circumstances (e.g., seeking social support from others and engaging in physical activities) in coping with stressors. Many public venues, such as public sports facilities and cultural centers, closed down during the pandemic, which also led to the reduction of activities (103, 104). Therefore, our results suggest that patients who are more dependent on external conditions need more help in coping with stressors, one of the approaches being the use of internal-driven active coping strategies, such as positive reappraisal and problem-solving-oriented strategies. Certainly, the whole point of doing this is to help them increase their PA.

### Limitations

Despite the strength of this study, it still has some limitations. First, this is a cross-sectional study; thus, the causality of the variables could not be reflected. Longitudinal studies are needed to find the causal relationship between the variables and PA in patients with SUDs. Second, this study is retrospective, and the data for analyses are from self-reports of patients, which might be subjective and limit the generalization of the results. Finally, female patients were not included in this study as female patients in the drug rehabilitation center only accounted for a very small portion at the time of our survey; thus, the gender balance was difficult to achieve with female patients included. Therefore, gender differences in PA of patients with SUDs need to be explored in future works.

## Conclusions

In summary, this study explored the differences and factors of PA between patients with HUD and patients with MAUD during the COVID-19 epidemic. Patients with SUDs are both physically and mentally vulnerable to such infectious diseases and therefore need attention from healthcare providers.

## Data Availability Statement

The raw data supporting the conclusions of this article will be made available by the authors, without undue reservation.

## Ethics Statement

The studies involving human participants were reviewed and approved by Ethics Committee of the Second Xiangya Hospital of Central South University. The patients/participants provided their written informed consent to participate in this study.

## Author Contributions

TL and XZ designed and supervised this study. YW, LW, XW, and QY collected data. QW collated the raw data. JZ analyzed and interpreted the data. YW wrote the first draft of the manuscript. XZ, DW, HW, and CG discussed and revised the manuscript. All co-authors approved the version to be published.

## Funding

This study was supported by the National Natural Science of China (Grant No. 81371465 and 81671324), the National Key R&D Program of China (2017YFC1310400), and the provincial Natural Science Foundation of Hunan (Grant No. 2015JJ2180).

## Conflict of Interest

The authors declare that the research was conducted in the absence of any commercial or financial relationships that could be construed as a potential conflict of interest.

## Publisher's Note

All claims expressed in this article are solely those of the authors and do not necessarily represent those of their affiliated organizations, or those of the publisher, the editors and the reviewers. Any product that may be evaluated in this article, or claim that may be made by its manufacturer, is not guaranteed or endorsed by the publisher.
